# Dissecting and tracing the gut microbiota of infants with botulism: a cross sectional and longitudinal study

**DOI:** 10.3389/fmicb.2024.1416879

**Published:** 2024-05-31

**Authors:** Dai Wang, Kexin Li, Lijuan Wang, Zhongqiu Teng, Xia Luo, Hui Sun, Ying Huang, Songnian Hu, Xuefang Xu, Zilong He

**Affiliations:** ^1^Xiamen Key Laboratory of Perinatal-Neonatal Infection, Xiamen Women and Children's Hospital, Department of Pathology, State Key Laboratory of Molecular Vaccinology and Molecular Diagnostics, Department of Laboratory Medicine, School of Public Health, Xiamen University, Xiamen, Fujian, China; ^2^School of Engineering Medicine, Beihang University, Beijing, China; ^3^Beijing Advanced Innovation Center for Big Data-Based Precision Medicine, Interdisciplinary Innovation Institute of Medicine and Engineering, Beihang University, Beijing, China; ^4^State Key Laboratory of Microbial Resources, Institute of Microbiology, Chinese Academy of Sciences, Beijing, China; ^5^Pediatric Intensive Care Unit, Beijing Children’s Hospital, National Center for Children's Health, Capital Medical University, Beijing, China; ^6^National Key Laboratory of Intelligent Tracking and Forecasting for Infectious Diseases and National Institute for Communicable Diseases Control and Prevention, Chinese Center for Disease Control and Prevention, Beijing, China

**Keywords:** infant botulism, 16S rRNA sequencing, whole genome sequencing, *Bifidobacterium*, longitudinal study

## Abstract

**Background:**

Infant botulism is caused by botulinum neurotoxin (BoNT), which is mainly produced by *Clostridium botulinum*. However, there is a lack of longitudinal cohort studies on infant botulism. Herein, we have constructed a cross-sectional and longitudinal cohort of infants infected with *C. botulinum*. Our goal was to reveal the differences in the intestinal microbiota of botulism-infected and healthy infants as well as the dynamic changes over time through multi-omics analysis.

**Methods:**

We performed 16S rRNA sequencing of 20 infants’ stools over a period of 3 months and conducted whole genome sequencing of isolated *C. botulinum* strains from these laboratory-confirmed cases of infant botulism. Through bioinformatics analysis, we focused on the changes in the infants’ intestinal microbiota as well as function over time series.

**Results:**

We found that *Enterococcus* was significantly enriched in the infected group and declined over time, whereas *Bifidobacterium* was significantly enriched in the healthy group and gradually increased over time. 18/20 isolates carried the type B 2 botulinum toxin gene with identical sequences. *In silico* Multilocus sequence typing found that 20\u00B0*C. botulinum* isolates from the patients were typed into ST31 and ST32.

**Conclusion:**

Differences in intestinal microbiota and functions in infants were found with botulism through cross-sectional and longitudinal studies and *Bifidobacterium* may play a role in the recovery of infected infants.

## Introduction

Infant botulism is one of the 6 forms of botulism (infant botulism, foodborne botulism, wound botulism, adult intestinal colonization, iatrogenic botulism, and inhalation botulism) (Shirey et al., 2015). Botulism is caused by botulinum neurotoxin (BoNT), which is mainly produced by *Clostridium botulinum* and rarely, by neurotoxigenic *Clostridium baratii* or *Clostridium butyricum* (Johnson et al., 1979). The illness is usually found in infants less than 1 year of age. *C. botulinum*, *C. baratii*, *C. butyricum* and *C. sporogenes* (BoNT-producing *Clostridia*) are responsible for infant and adult intestinal colonization botulism and can germinate, reproduce, and release BoNTs into the gut. The toxin is absorbed via lymphatic into the blood and transported to the neuromuscular junction, where it binds with soluble N-ethylmaleimide-sensitive factor attachment protein receptor, blocks acetylcholine release, and leads to flaccid paralysis (Arnon et al., 2006; Payne et al., 2018). Infant botulism is the major form of botulism in the United States (CDC, 2012) and has been found across the world (Drivenes et al., 2017; Dilena et al., 2021; Jeon et al., 2021; Avila et al., 2022; Douillard et al., 2022; Nga et al., 2022; Oliveira et al., 2022; Goldberg et al., 2023). Infant botulism was first reported in China in 1990 (Wu et al., 1990) and more cases have been reported in recent years with better diagnostic technologies (Xin et al., 2019; Ge et al., 2020; Zhang et al., 2022).

There are a few studies on the infection source and epidemiology of infant botulism (Koepke et al., 2008; Gladney et al., 2021). Some studies showed that BoNT-producing *Clostridia* spores are prevalent in soil which is the most likely source of infant botulism. A recent study in Colorado, United States found that the isolates from infant botulism cases were classified into a cluster from soil and dust, indicating a close relationship in genome sequences and a possible source from soil (Gladney et al., 2021). Infant botulism is known to affect the gut microbiota. Shirey TB et al. found significant differences in *Proteobacteria*, *Firmicutes*, and *Enterobacteriaceae* abundances in the fecal microbiota of infants with botulism compared to non-confirmed cases samples (Shirey et al., 2015). However, there is a lack of longitudinal cohort studies on gut microbiota variation in infant botulism in the short and long term, especially in gut microbiota following recovery.

In this study, we constructed a cross-sectional and longitudinal cohort of infants infected with *C. botulinum*. Stool samples for 16S rRNA sequencing were collected from 1 to 90 days of confirmed infected infants. Our goal was to reveal the differences in the intestinal microbiota and metabolism changes of botulism-infected infants compared with healthy infants as well as the dynamic changes over time through multi-omics analysis following treatment. The aim of the study was to gain a better understanding of the changes in intestinal microbiota caused by infant botulism and during recovery, to provide a better theoretical basis for the treatment of the disease.

## Materials and methods

### Sample collection

This study was conducted in accordance with the guidelines of the Helsinki Declaration and Rules of Good Clinical Practice. In compliance with human subjects’ exemption protocol (SHERLL 2019057) approved by the Ethics Committee of the Capital Institute of Pediatrics. Stool samples for 16S rRNA gene sequencing were collected from 20 infants who were all confirmed as BoNT gene positive by quantitative PCR from 2015 to 2021 (Huang et al., 2019). In addition, 10 stool samples were obtained from 10 healthy infants aged from 2 to 8 months for 16S rRNA gene sequencing analysis as BoNT gene negative by quantitative PCR. Besides, at least one botulism isolate from each patient was cultured and sequenced. Detailed meta information for 16S rRNA gene sequencing samples and isolated strains is listed in Table 1.

**Table 1:** Metadata of infant cohort.

Samples	Age	Sex	Location	Sampling date after Infection	Individuals isolates	Neurotoxin typing
C1	8	Male	Beijing	0		
C2	2	Male	Beijing	0		
C3	4	Male	Beijing	0		
C4	7	Female	Beijing	0		
C5	5	Male	Beijing	0		
C6	3	Female	Beijing	0		
C7	4	Male	Beijing	0		
C8	6	Female	Hebei	0		
C9	5	Female	Hebei	0		
C10	5	Male	Hebei	0		
I1	6	Female	Shandong	0,7	*	BoNT B2
I2	3	Male	Shandong	0,30	*	BoNT B2
I3	6	Female	Shanxi	0	*	BoNT B2
I4	4	Male	Hebei	0	*	BoNT B2
I5	5	Male	Hebei	0,7,14,30,60	*	BoNT B2
I6	3	Male	Beijing	0,30,60,90	*	N/A
I7	8	Male	Beijing	0,7,14,30	*	N/A
I8	6	Female	Beijing	0,7,14	*	BoNT B2
I9	7	Female	Hebei	0	*	BoNT B2
I10	4	Female	Hebei	0	*	BoNT B2
I11	4	Male	Hebei	1,7,14	*	BoNT B2
I12	7	Female	Hebei	0	*	BoNT B2
I13	2	Female	Shandong	0	*	BoNT B2
I14	3	Male	Beijing	0,14	*	BoNT B2
I15	8	Female	Shanxi	0	*	BoNT B2
I16	3	Female	Shandong	0,7,30,60	*	BoNT B2
I17	5	Male	Beijing	0,14,30	*	BoNT B2
I18	7	Male	Hebei	0,30,60,90	*	BoNT B2
I19	6	Male	Shandong	0,7,14	*	BoNT B2
I20	4	Female	Shanxi	0	*	BoNT B2

C stands for healthy infants. I stands for infant botulism infants. * stands for positive results.

### DNA extraction and sequencing

All the stool samples for 16S rRNA sequencing were collected and frozen at −80°C after sampling. DNA was extracted using the E.Z.N.A stool DNA kit as the manufacturer’s instructions for 16S rRNA sequencing. The 16S rRNA gene were amplified for PCR amplification using universal primers 341F (5’-CCTAYGGGRBGCASCAG-3′) and 806R (3’-GGACTACNNGGGTATCTAAT-5′). According to the manufacturer’s instructions, the library was constructed using TruSeq DNA PCR-free sample preparation kit. Each PCR reaction was comprised of 5 μL of 10 × PCR buffer, 0.5 μL of dNTP (10 mM), 0.5 μL of PCR forward primer (50 μM), 0.5 μL of revese primer (50 μM), 0.5 μL of Platinum Taq (5 U/μL) (Thermo Fisher Scientific, USA) and 20 ng DNA in a total volume of 50 μL. PCR conditions comprised of initial denaturation at 95°C for 5 min, followed by 29 cycles of denaturation at 94°C for 30 s, annealing at 50°C for 60 s and elongation at 72°C for 60 s, with the final extension at 72°C for 7 min. Each sample was indexed and sequenced using the Illumina MiSeq PE-300. Pure strains of *C. botulinum* isolated from laboratory-confirmed cases of infant botulism were submitted to DNA extraction, and the total DNA of these isolated strains was purified from overnight culture using Qiagen Genomic-tip 100/G columns (Qiagen, Germantown, MD, United States) under the manufacturer’s instructions. The purity and integrity of the DNA were tested by agarose gel electrophoresis and quantified by Qubit. Qualified DNA samples detected by electrophoresis were randomly broken into fragments. Libraries were prepared using a Nextera XT DNA library preparation kit (Illumina, Inc., Cambridge, UK). Whole genome sequencing (WGS) was performed using Illumina NovaSeq 6,000 with PE-150 model.

### Processing of 16S rRNA sequencing data

All 16S rRNA sequencing data were processed with the Quantitative Insights into Microbial Ecology version 2 (QIIME2) software (Bolyen et al., 2019). The DADA2 plugin was used for quality control, filtering chimeric sequences, and assembling reads (Callahan et al., 2016). Q2-feature-classifier plugin with the Greengene database was used to do taxonomic annotation (DeSantis et al., 2006). Alpha diversity and Principal Coordinates Analysis (PCoA) based on Bray-Curtis diversity were performed by vegan and ggplot2 package in R software (version 3.6.1). The LEfSe was applied to determine the microbial taxa with significantly differential abundance between groups (LDA > 3.0) (Segata et al., 2011). Metabolic pathways were predicted by PICRUSt2 using the KEGG database (Douglas et al., 2020). The co-abundance networks of microbial taxa were visualized by Cytoscape version 3.72.

### Processing of whole genome sequencing data

Low quality and adapter reads were removed by trimmomatic v0.39. The reads were assembled using SPAdes v3.14.0 with the “- careful” option (Prjibelski et al., 2020). QUAST v4.6 and BUSCO v5 were used to conduct a quality check of the genomes and infer the annotation completeness (Gurevich et al., 2013; Manni et al., 2021). Prokka v1.13 was used to predict and annotate the genome assemblies (Seemann, 2014). The pan genome analysis was performed using Roary v3.13 with default parameters (Page et al., 2015). Multilocus sequence typing (MLST) schemes were determined using the PubMLST *C. botulinum* database.^1^ The multi-sequence alignment was performed by mafft and visualized by MEGA X (Katoh et al., 2002; Kumar et al., 2018).

### Statistical analysis

R scripts with packages ggplot2 and vegan in R v3.6.1 were used for statistical analysis and visualization. The heatmap was drawn by the pheatmap package.

## Result

### The overview of infant cohorts and sequencing data

Stool samples were collected from a total of 30 infants for the study (10 healthy and 20 infected infants). The 30 infants came from northern cities in China and ranged in age from 2 months to 8 months, with a sex ratio of nearly 1:1 (Table 1). To investigate the ongoing effects of *C. botulinum* on infant gut microbiota, we collected the fecal samples from 12 of 20 infected infants at days 1, 7, 14, 30, 60 and 90. A total of 47 longitudinal cohort samples were collected for 16S rRNA analysis. On average, each 16S sample contained 71,427 ± 11,050 reads across all samples. In addition, 20 strains of *C. botulinum* were isolated from infected infant fecal samples for whole genome sequencing. The total size of whole genomes sequencing for strains was 26.46 G, with an average sequencing coverage of 339 ± 122.36 × per strain. Following pre-processing, all samples were saturated by genome completion assessment and ready for subsequent analysis. The specific analysis flow is shown in Supplementary Figure S1.

### The composition of intestinal microbiota in healthy and *Clostridium botulinum*-infected infants

**Figure 1:**
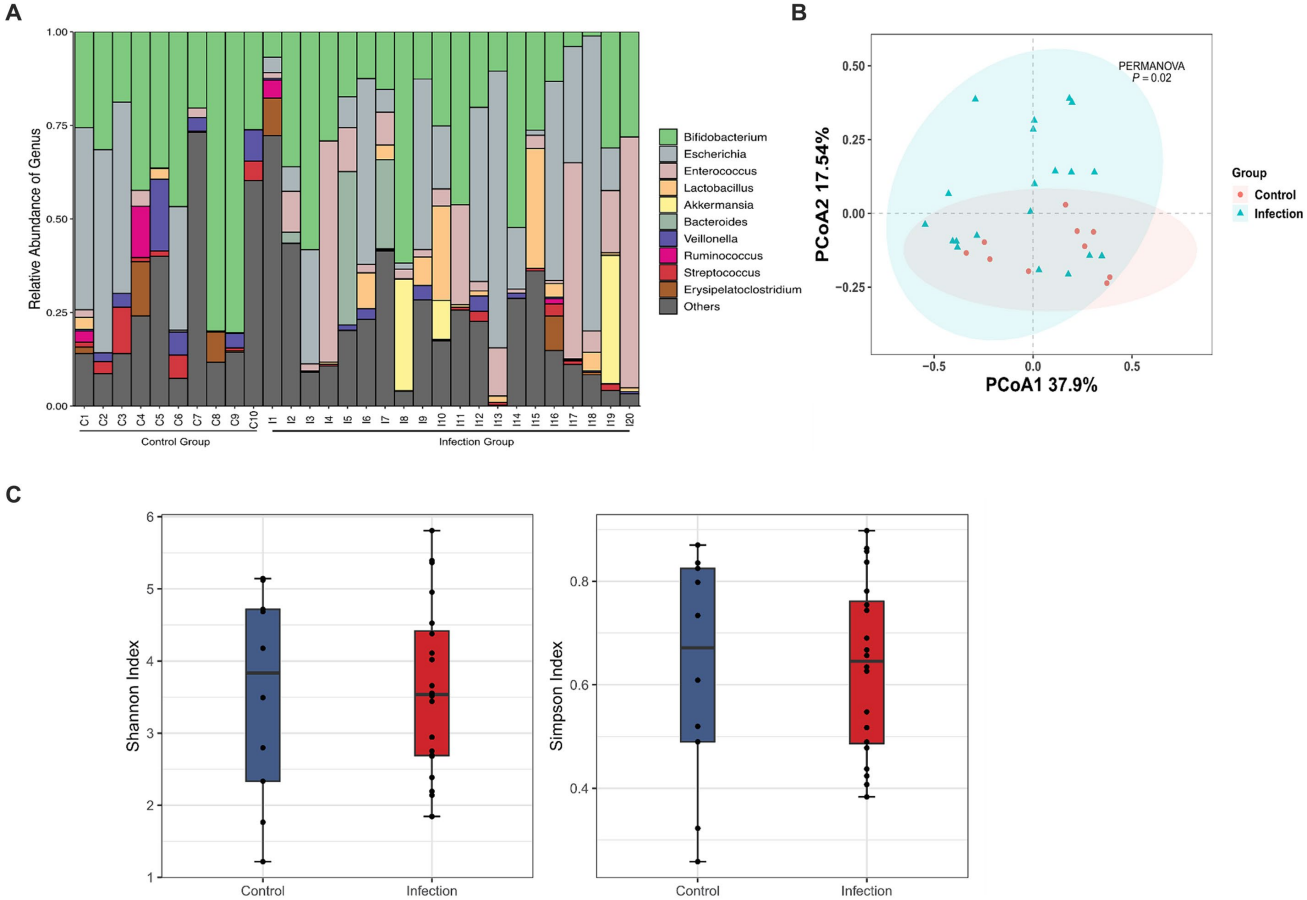
Diversity analysis based on 16S rRNA analysis. **(A)** Taxonomic classification at the genus level between control and infection groups (day1). **(B)** Principal coordinate analysis plot between control and infection groups based on Bray Curtis of bacterial communities (PERMANOVA, FDR, *p* < 0.05). **(C)** Shannon and Simpson indexes between Control (red) and Infection (blue) samples.

Based on the standard 16S data analysis protocol, 16 phyla, 24 orders, 34 orders, 54 families, 138 genera and 195 species were identified in healthy and *C. botulinum* -infected infants. In terms of taxonomic composition, the top 3 phyla were *Actinobacteriota* (37.46% mean abundance), *Firmicutes* (29.65%) and *Proteobacteria* (26.20%). At the genus level (Figure 1A), the most abundant genera were *Bifidobacterium* (31.58%), *Escherichia* (15.87%) and *Enterococcus* (10.24%). In terms of diversity, differences in the overall distribution between the healthy and infected groups were found using PCoA analysis based on the Bray–Curtis dissimilarity method (PERMANOVA, *p* value <0.05) (Figure 1B). The Shannon index and the Simpson index of the healthy group were higher than those of the infected group, but the difference was not statistically significant (Figure 1C).

### The classification and functional differences in intestinal microorganisms between healthy and *Clostridium botulinum* infected infant cohorts

**Figure 2:**
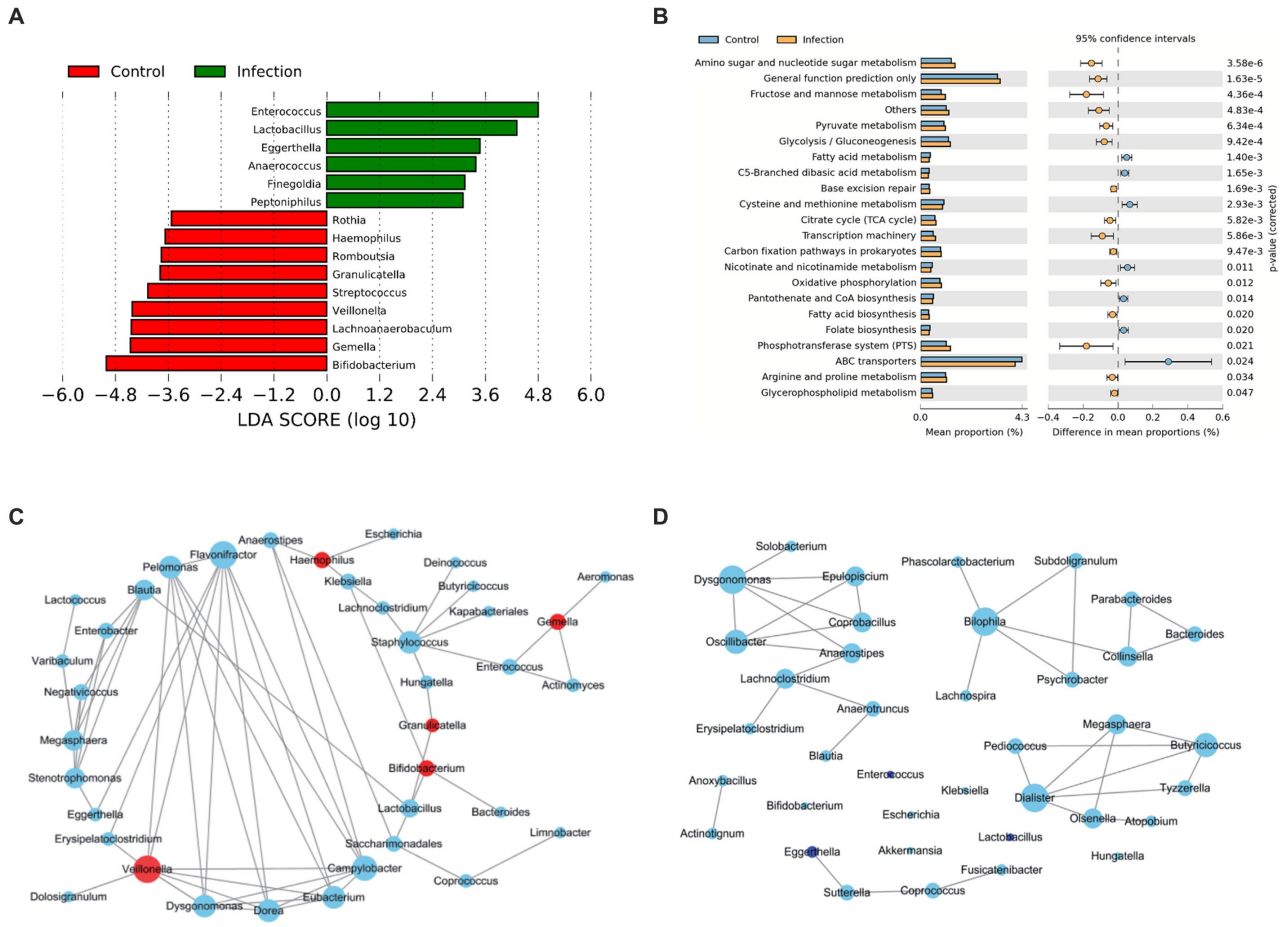
Bacteria analysis and functional categories of the bacterial communities predicted by PICRUSt2 analysis. **(A)** Lefse analysis at the genus level between control and infection groups (Wilcox test, FDR, *p* < 0.05). **(B)** Functional categories of the differentiated pathways at KEGG level 3 between control and infection groups (Welch’s t-test, *p* < 0.05). The bars in the graph represent the mean proportion (%) of the functional categories. The 95% confidence intervals reflect the difference in mean proportions (%), and corrected *p* values are displayed. **(C,D)** Co-abundance networks of genera between control **(C)** and infection **(D)** groups. The red dots represent the differentially abundant genera for each group. The blue dots represent intestinal genera that were not differentially abundant genera. The size of the dots represents the degree. The links represent the interactions of genera. The correlations with *r* > 0.75 and *p* < 0.05 are shown.

To systematically assess the differences in intestinal microbiota between healthy and infected infants, we analyzed the microbial communities with different abundance and the function of differential microorganisms. We detected 15 differentially abundant microbiotas at the genus level (LDA > 2), with *Bifidobacterium* showing the greatest degree of difference among those with higher abundance in the healthy group (LDA > 4). *Gemella*, *Lachnoanaerobaculum,* and *Veillonella* were also different in the two groups. Besides, differential abundant microbiota with higher abundance in the infected group were *Enterococcus* (LDA > 4) and *Lactobacillus* (LDA > 3) (Figure 2A).

In terms of tertiary metabolic functions, the two groups differed in 21 pathways (*p* < 0.05) (Figure 2B). The metabolic pathways with the greatest differences were mainly amino sugar and nucleotide sugar metabolism, fructose and mannose metabolism, and pyruvate metabolism, which were significantly enriched in the infected group. Compared with the control group, fatty acid metabolism, cysteine and methionine metabolism, and nicotinate and nicotinamide metabolism decreased in the infection group.

In addition, we constructed microbial co-abundance networks for the healthy group and the infected group of infants, respectively, (Figures 2C,D). We found that the network in the healthy group was more tightly linked than the network in the infected group, with more connections between nodes. In the healthy group, the degree of all the differential abundant microbiota was less than or equal to 3, except for *Veillonella*, which had a linkage greater than 5. *Flavonifractor* (degree = 8) was the most linked taxon in the healthy group network (Figure 2C). The overall degree of the infected group network was not high. The most linked taxa in the infected group network were *Dysgonomonas*, *Bilophila*, and *Dialister*, all of which had a linkage of 5 (Figure 2D).

### Longitudinal analysis reveals the dynamics of key microbiota from *Clostridium botulinum* infected infants

**Figure 3:**
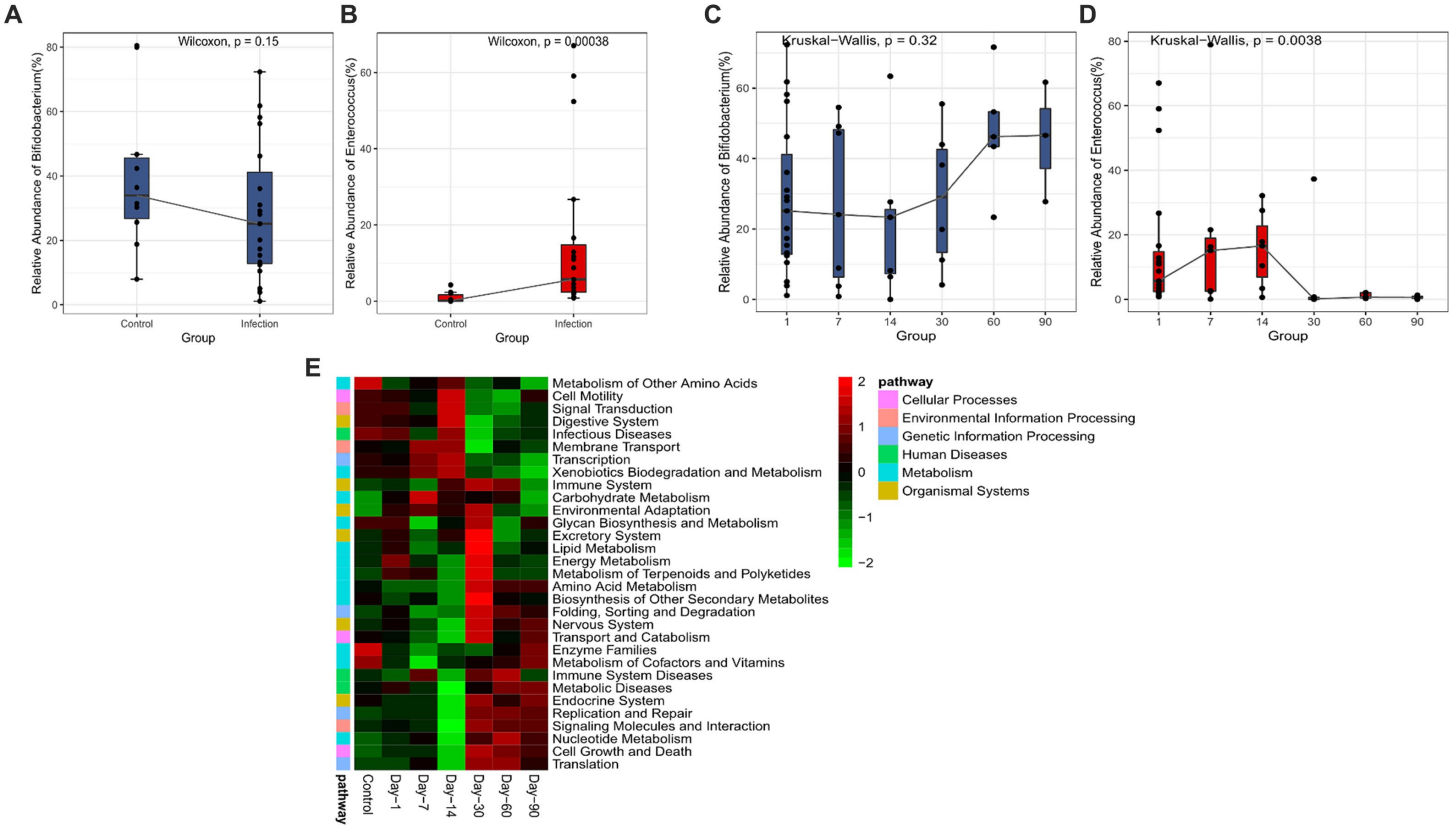
Analysis of significant bacteria and functional categories in different groups. **(A)** *Bifidobacterium* in Control and Infection groups. **(B)** *Enterococcus* in Control and Infection groups. **(C)** Changing of *Bifidobacterium* in days 1, 7, 14, 30, 60, 90. **(D)** Changing of Enterococcus in day 1, 7, 14, 30, 60, 90. **(E)** Heatmap of the relative abundances of pathways in different groups predicted by PICRUSt2 analysis at KEGG level 3 based on z-scores.

In the differential abundant microbiota analysis described above, we found that *Bifidobacterium* and *Enterococcus* differed most in the two cohorts. To examine the temporal dynamics of these two groups, we followed up on some of the infected infants (12/20) and collected stool samples for the longitudinal study. The cross-sectional 16S data analysis also showed significant differences in *Enterococcus* (*p* < 0.05) (Figures 3A,B). The overall abundance of *Bifidobacterium* increased significantly after 30 days and the dispersion throughout decreased, whereas *Enterococcus* decreased rapidly after 14 days and was almost undetectable after 30 (Figures 3C,D). We also compared the samples of patients at the initial stage of infection (Infection group) with the samples of patients at 60 days and 90 days after disease diagnosis (Recovery group). We found that the differential abundant bacteria between the two groups were also different between the infected group and the healthy group, except for *Bifidobacterium* (Supplementary Figure S2). Secondary metabolic pathway analysis revealed that the microbial-related metabolic pathways like Glycan biosynthesis and metabolism, amino acid metabolism, metabolism of cofactors and vitamins, and biosynthesis of other secondary metabolites in the infected group gradually returned to the levels of the control group over time (Figure 3E).

### Genomic analysis of *Clostridium botulinum* isolates from infected infants

The 20 *C. botulinum* isolates from the stool samples of the 20 infected infants with one isolate per case were sequenced using Illumina sequencing. The average genome length ranged from 3.8 to 4.3 M, with an average N50 of around 846 kb (Supplementary Table S1). 18/20 isolates carried the botulinum toxin gene with identical nucleotide sequence encoding the type B 2 botulinum toxin (Supplementary Figure S3). *In silico* MLST typing found that the 20 *C. botulinum* isolates from the patients were typed into two sequence types (ST), ST31 and ST32 with 10 isolates each. KEGG enrichment analysis based on the core genome of 20 strains of *C. botulinum* showed that ABC transporters and two-component system pathways had the highest gene enrichment degree (Supplementary Figure S4).

## Discussion

The initial sterile gut in the infant is an oxidized environment favorable to be colonized by facultative aerobes such as *Lactobacillus*, *Prevotella,* and *Sneathia* sp. With less oxygen in the gut, it becomes more suitable for anaerobic bacteria to grow (Penders et al., 2006). Therefore, BoNT-producing *Clostridia* infection mostly occurs in infants aged 0 to 8 months. Because the intestinal microbiota of infants is not stable, it is likely to carry spores into the gut through the mouth, thus causing poisoning. At present, there are few studies on the intestinal microbiota of infants with botulism. Only one study in the US reported intestinal microbial profiling of 14 infected infants (Shirey et al., 2015). Most botulinum toxin studies are associated with strains isolated from humans or the environment, but there is a lack of cohort comparison in cross-sectional and longitudinal analysis of intestinal microbiota in infant botulism. In this study, we first collected the fecal samples of infants infected with *C. botulinum* from China. In addition, some of the isolates were isolated from infant feces and the surrounding environment. To study the dynamic changes of intestinal microbiota in infants infected with *C. botulinum* on a time scale, we tracked and collected fecal samples from some infants at multiple time points. The strategy of cross-sectional and longitudinal study based on metagenome and whole genome sequencing of bacteria may help us better understand the infection process and mechanism of botulism.

Shirey et al. examined 14 infant fecal samples including 8 botulism samples and 6 non-confirmed which were used as control (Shirey et al., 2015). Due to the lack of metadata on the samples, the non-confirmed samples might not be strictly healthy controls. In this study, we investigated 20 botulism samples which were not only confirmed by PCR but also by strain isolation. 10 healthy infant fecal samples were collected as control covering all the age spectrum of the confirmed samples. Shirey et al. illustrated that the abundance of *Enterobacteriaceae* in infant intestinal microbiota of botulism is significantly increased, which is consistent with our findings (Shirey et al., 2015). We found that the abundance of *Escherichia* and *Enterococcus* were all increased in the infected group compared with the healthy group. In the time scale analysis, we also found that the abundance of *Enterococcus* in the infected group gradually decreased after 14 days until it could not be detected, indicating that *Enterococcus* may be involved in the occurrence of the infection. Interestingly, it was also reported an increase in *Enterococcus* in children infected with *Clostridioides difficile* (Ling et al., 2014). In addition, the LDA score of *Bifidobacterium* was very high in the healthy group compared with the infected group. It is well known that *Bifidobacterium*, as a probiotic, plays an important role in the regulation of intestinal microbiome (Stuivenberg et al., 2022). Many studies have reported that *Bifidobacterium* plays a positive role in promoting intestinal health (O'Callaghan and van Sinderen, 2016; Turroni et al., 2019; Derrien et al., 2022). In our study, the abundance of *Bifidobacterium* in the healthy group was higher than that in the infected group (LDA > 4). The time scale analysis showed that the abundance of *Bifidobacterium* increased significantly after 30 days in the infection group, indicating that the abundance of *Bifidobacterium* decreased after the initial infection, and increased during the recovery of infection. This phenomenon is consistent with previous reports indicating that infected children progressively return to a “healthy microbiota status” (Becker-Dreps et al., 2015; Singh et al., 2015). Hence, it may be indicated that the supplement of *Bifidobacterium* during the infection process may help in the treatment of the infection. Furthermore, metabolic changes caused by microflora changes were also studied here. Amino acid metabolism including arginine, proline, cysteine, and methionine were altered. We know that high protein concentration was needed in the growth of *C. botulinum*. Therefore, we infer that these amino acid changes are related to *C. botulinum* growth.

In this study, genomic analysis of *C. botulinum* isolates identified two STs, neither of which has been reported in previous studies. Previous studies have reported genotyping of *C. botulinum* from infants to be predominantly ST2, with fewer strains of other ST typing (Brunt et al., 2020). In terms of botulinum toxin identification, 18/20 strains were found to carry the botulinum toxin gene. Interestingly, all the botulinum toxin genes identified were type B 2 and the protein sequences were highly consistent, suggesting that the botulinum toxins prevalent in northern China are likely to be closely related as previously reported (Vanhomwegen et al., 2013; Ma et al., 2022). What’s more, we downloaded 68 complete-level genome sequences of *C. botulinum* in NCBI database. We found B5 was the most toxin subtype (*n* = 23), followed by A1 (*n* = 22), A2 (*n* = 12), and, as reported here, B2 (*n* = 10) (Supplementary Figure S5).

In this project, we used a strategy of 16S sequencing and whole genome sequencing of single bacteria isolates to study *C. botulinum* infection. Unfortunately, 16S rRNA sequencing failed to obtain OTUs of *C. botulinum*, which may be due to the resolution of the 16S segments and thus the inability to accurately quantify the abundance of *C. botulinum*. Given the low microbial content of infant feces, the full-length 16S rRNA sequencing based on third-generation sequencing may be more suitable. This technology can pinpoint the species-level abundance of most microorganisms without requiring much raw DNA volume compared to normal 16S rRNA sequencing, which may be a better choice for the study of intestinal microbes in infants in the future. In addition, a combined strategy of full-length 16S sequencing and whole genome sequencing is also recommended.

## Conclusion

In conclusion, we have systematically analyzed the differences in gut microbes and functions in botulism-affected infants through cross-sectional and longitudinal studies. We found that *Enterococcus* was significantly enriched in the infected group and declined over time, whereas *Bifidobacterium* was significantly enriched in the healthy group and gradually increased over time following infection in infected infants. Pathway analysis revealed that the metabolism of cofactors and vitamins, and amino acid metabolism gradually returned to normal levels over time. The findings provided a better understanding of the changes in intestinal microbiota caused by infant botulism and during recovery and a better theoretical basis for the management of the disease.

## Data availability statement

Sequencing data were deposited in NCBI with accession numbers PRJNA908234 and PRJNA931660.

## Ethics statement

The studies involving humans were approved by this study was conducted in accordance with the guidelines of the Helsinki Declaration and Rules of Good Clinical Practice. In compliance with human subjects’ exemption protocol (SHERLL 2019057) approved by Ethics Committee of the Capital Institute of Pediatrics. The studies were conducted in accordance with the local legislation and institutional requirements. Written informed consent for participation in this study was provided by the participants' legal guardians/next of kin.

## Author contributions

DW: Writing – review & editing, Writing – original draft, Supervision, Software, Investigation, Funding acquisition, Data curation, Conceptualization. KL: Writing – review & editing, Writing – original draft, Software, Methodology, Investigation, Formal analysis, Data curation. LW: Writing – original draft, Resources. ZT: Writing – original draft, Methodology, Formal analysis, Data curation. XL: Writing – original draft, Methodology, Investigation. HS: Writing – original draft, Validation. YH: Writing – original draft, Validation. SH: Writing – review & editing, Resources. XX: Writing – review & editing, Writing – original draft, Validation, Supervision, Project administration, Methodology, Investigation, Formal analysis, Data curation, Conceptualization. ZH: Writing – review & editing, Writing – original draft, Validation, Supervision, Software, Methodology, Investigation, Formal analysis, Data curation, Conceptualization.

## Footnotes

^1^ http://pubmlst.org/cbotulinum/

## References

[ref1] Arnon S. S. Schechter R. Maslanka S. E. Jewell N. P. Hatheway C. L. (2006). Human botulism immune globulin for the treatment of infant botulism. N. Engl. J. Med. 354, 462–471. doi: 10.1056/NEJMoa051926 16452558

[ref2] Avila C. E. Cardenas M. M. Kaltenbach G. H. Lazzarini L. Pierangeli N. (2022). Infant botulism: a descriptive study in a pediatric intensive care unit. Arch. Argent. Pediatr. 121:e202202656. doi: 10.5546/aap.2022-02656.eng 36413169

[ref3] Becker-Dreps S. Allali I. Monteagudo A. Vilchez S. Hudgens M. G. Rogawski E. T.. (2015). Gut microbiome composition in young Nicaraguan children during diarrhea episodes and recovery. Am. J. Trop. Med. Hyg. 93, 1187–1193. doi: 10.4269/ajtmh.15-0322, PMID: 26350452 PMC4674233

[ref4] Bolyen E. Rideout J. R. Dillon M. R. Bokulich N. A. Abnet C. C. Al-Ghalith G. A.. (2019). Author correction: reproducible, interactive, scalable and extensible microbiome data science using QIIME 2. Nat. Biotechnol. 37:1091. doi: 10.1038/s41587-019-0252-6, PMID: 31399723

[ref5] Brunt J. van Vliet A. H. M. Carter A. T. Stringer S. C. Amar C. Grant K. A.. (2020). Diversity of the genomes and neurotoxins of strains of *Clostridium botulinum* group I and *Clostridium sporogenes* associated with foodborne, infant and wound botulism. Toxins 12:586. doi: 10.3390/toxins12090586, PMID: 32932818 PMC7551954

[ref6] Callahan B. J. McMurdie P. J. Rosen M. J. Han A. W. Johnson A. J. Holmes S. P. (2016). DADA2: high-resolution sample inference from Illumina amplicon data. Nat. Methods 13, 581–583. doi: 10.1038/nmeth.3869, PMID: 27214047 PMC4927377

[ref7] CDC. (2012). National botulism surveillance system. Available at: http://www.cdc.gov/nationalsurveillance/botulism-surveillance.html

[ref8] Derrien M. Turroni F. Ventura M. van Sinderen D. (2022). Insights into endogenous *Bifidobacterium* species in the human gut microbiota during adulthood. Trends Microbiol. 30, 940–947. doi: 10.1016/j.tim.2022.04.004, PMID: 35577716

[ref9] DeSantis T. Z. Hugenholtz P. Larsen N. Rojas M. Brodie E. L. Keller K.. (2006). Greengenes, a chimera-checked 16S rRNA gene database and workbench compatible with ARB. Appl. Environ. Microbiol. 72, 5069–5072. doi: 10.1128/AEM.03006-05, PMID: 16820507 PMC1489311

[ref10] Dilena R. Pozzato M. Baselli L. Chidini G. Barbieri S. Scalfaro C.. (2021). Infant botulism: checklist for timely clinical diagnosis and new possible risk factors originated from a case report and literature review. Toxins 13:860. doi: 10.3390/toxins13120860, PMID: 34941698 PMC8703831

[ref11] Douglas G. M. Maffei V. J. Zaneveld J. R. Yurgel S. N. Brown J. R. Taylor C. M.. (2020). PICRUSt2 for prediction of metagenome functions. Nat. Biotechnol. 38, 685–688. doi: 10.1038/s41587-020-0548-6 32483366 PMC7365738

[ref12] Douillard F. P. Derman Y. Woudstra C. Selby K. Maklin T. Dorner M. B.. (2022). Genomic and phenotypic characterization of *Clostridium botulinum* isolates from an infant botulism case suggests adaptation signatures to the gut. MBio 13:e0238421. doi: 10.1128/mbio.02384-21, PMID: 35499308 PMC9239077

[ref13] Drivenes B. Krause T. G. Andersson M. Muller L. Fuursted K. Pedersen T.. (2017). Infant botulism in Denmark from 1995 to 2015. Dan. Med. J. 64:A5404. 28874241

[ref14] Ge X. S. Xu Q. Liu X. Huang S. Gao Y. Liu P.. (2020). Clinical analysis and laboratory diagnosis of three cases with infantile botulism caused by *Clostridium botulinum* type B. Chin. J. Pediatr. 58, 499–502. doi: 10.3760/cma.j.cn112140-20191101-00691 C 32521963

[ref15] Gladney L. Halpin J. L. Luquez C. (2021). Genomic characterization of strains from a cluster of infant botulism type a in a small town in Colorado, United States. Front. Microbiol. 12:688240. doi: 10.3389/fmicb.2021.688240, PMID: 34326824 PMC8313963

[ref16] Goldberg B. Danino D. Levinsky Y. Levy I. Straussberg R. Dabaja-Younis H.. (2023). Infant Botulism, Israel, 2007-2021. Emerg. Infect. Dis. 29, 235–241. doi: 10.3201/eid2902.220991, PMID: 36692296 PMC9881770

[ref17] Gurevich A. Saveliev V. Vyahhi N. Tesler G. (2013). QUAST: quality assessment tool for genome assemblies. Bioinformatics 29, 1072–1075. doi: 10.1093/bioinformatics/btt086, PMID: 23422339 PMC3624806

[ref18] Huang Y. Shi Y. Ye C. Xu X. (2019). Establishment of real-time PCR assays for rapid detection of *Clostridium botulinum* type a and B. Dis. Surveill. 34, 844–848. doi: 10.3784/j.issn.1003-9961.2019.09.015

[ref19] Jeon J. H. Choi C. H. Kim J. H. Hyun J. Choi E. S. Choi S. Y.. (2021). Genetic characterization of *Clostridium botulinum* isolated from the first case of infant botulism in Korea. Ann. Lab. Med. 41, 489–492. doi: 10.3343/alm.2021.41.5.489, PMID: 33824238 PMC8041589

[ref20] Johnson R. O. Clay S. A. Arnon S. S. (1979). Diagnosis and management of infant botulism. Am. J. Dis. Child. 133, 586–593, PMID: 375717 10.1001/archpedi.1979.02130060026004

[ref21] Katoh K. Misawa K. Kuma K. Miyata T. (2002). MAFFT: a novel method for rapid multiple sequence alignment based on fast Fourier transform. Nucleic Acids Res. 30, 3059–3066. doi: 10.1093/nar/gkf436, PMID: 12136088 PMC135756

[ref22] Koepke R. Sobel J. Arnon S. S. (2008). Global occurrence of infant botulism, 1976-2006. Pediatrics 122, e73–e82. doi: 10.1542/peds.2007-1827, PMID: 18595978

[ref23] Kumar S. Stecher G. Li M. Knyaz C. Tamura K. (2018). MEGA X: molecular evolutionary genetics analysis across computing platforms. Mol. Biol. Evol. 35, 1547–1549. doi: 10.1093/molbev/msy096, PMID: 29722887 PMC5967553

[ref24] Ling Z. Liu X. Jia X. Cheng Y. Luo Y. Yuan L.. (2014). Impacts of infection with different toxigenic *Clostridium difficile* strains on faecal microbiota in children. Sci. Rep. 4:7485. doi: 10.1038/srep07485, PMID: 25501371 PMC4265774

[ref25] Ma X. Li K. Li F. Su J. Meng W. Sun Y.. (2022). Tracing foodborne botulism events caused by *Clostridium botulinum* in Xinjiang Province, China, using a Core genome sequence typing scheme. Microbiol. Spectr. 10:e0116422. doi: 10.1128/spectrum.01164-22, PMID: 36377961 PMC9769928

[ref26] Manni M. Berkeley M. R. Seppey M. Zdobnov E. M. (2021). BUSCO: assessing genomic data quality and beyond. Curr. Protoc. 1:e323. doi: 10.1002/cpz1.323, PMID: 34936221

[ref27] Nga T. T. Hoang L. H. Trang L. T. Tram N. T. Yen P. B. Trung N. T.. (2022). First confirmed case of infant botulism caused by *Clostridium botulinum* type a(B) in a 10-month-old infant in Hanoi, Vietnam. IJID Reg. 5, 18–20. doi: 10.1016/j.ijregi.2022.08.003, PMID: 36147902 PMC9485906

[ref28] O'Callaghan A. van Sinderen D. (2016). Bifidobacteria and their role as members of the human gut microbiota. Front. Microbiol. 7:925. doi: 10.3389/fmicb.2016.00925 27379055 PMC4908950

[ref29] Oliveira L. M. Goncalves D. B. Cabral L. C. R. Bernardino M. R. A. Feitoza P. V. S. (2022). Botulism in the Brazilian Amazon: a life-threatening disease in a neglected population. Arq. Neuropsiquiatr. 80, 1227–1232. doi: 10.1055/s-0042-1758651, PMID: 36580960 PMC9800156

[ref30] Page A. J. Cummins C. A. Hunt M. Wong V. K. Reuter S. Holden M. T.. (2015). Roary: rapid large-scale prokaryote pan genome analysis. Bioinformatics 31, 3691–3693. doi: 10.1093/bioinformatics/btv421, PMID: 26198102 PMC4817141

[ref31] Payne J. R. Khouri J. M. Jewell N. P. Arnon S. S. (2018). Efficacy of human botulism immune globulin for the treatment of infant botulism: the first 12 years post licensure. J. Pediatr. 193, 172–177. doi: 10.1016/j.jpeds.2017.10.035, PMID: 29229452

[ref32] Penders J. Thijs C. Vink C. Stelma F. F. Snijders B. Kummeling I.. (2006). Factors influencing the composition of the intestinal microbiota in early infancy. Pediatrics 118, 511–521. doi: 10.1542/peds.2005-2824 16882802

[ref33] Prjibelski A. Antipov D. Meleshko D. Lapidus A. Korobeynikov A. (2020). Using SPAdes De novo assembler. Curr. Protoc. Bioinformatics 70:e102. doi: 10.1002/cpbi.102 32559359

[ref34] Seemann T. (2014). Prokka: rapid prokaryotic genome annotation. Bioinformatics 30, 2068–2069. doi: 10.1093/bioinformatics/btu153, PMID: 24642063

[ref35] Segata N. Izard J. Waldron L. Gevers D. Miropolsky L. Garrett W. S.. (2011). Metagenomic biomarker discovery and explanation. Genome Biol. 12:R60. doi: 10.1186/gb-2011-12-6-r60, PMID: 21702898 PMC3218848

[ref36] Shirey T. B. Dykes J. K. Luquez C. Maslanka S. E. Raphael B. H. (2015). Characterizing the fecal microbiota of infants with botulism. Microbiome 3:54. doi: 10.1186/s40168-015-0119-0, PMID: 26593441 PMC4655454

[ref37] Singh P. Teal T. K. Marsh T. L. Tiedje J. M. Mosci R. Jernigan K.. (2015). Intestinal microbial communities associated with acute enteric infections and disease recovery. Microbiome 3:45. doi: 10.1186/s40168-015-0109-2, PMID: 26395244 PMC4579588

[ref38] Stuivenberg G. A. Burton J. P. Bron P. A. Reid G. (2022). Why are Bifidobacteria important for infants? Microorganisms 10:278. doi: 10.3390/microorganisms10020278, PMID: 35208736 PMC8880231

[ref39] Turroni F. Duranti S. Milani C. Lugli G. A. van Sinderen D. Ventura M. (2019). *Bifidobacterium bifidum*: a key member of the early human gut microbiota. Microorganisms 7:544. doi: 10.3390/microorganisms7110544, PMID: 31717486 PMC6920858

[ref40] Vanhomwegen J. Berthet N. Mazuet C. Guigon G. Vallaeys T. Stamboliyska R.. (2013). Application of high-density DNA resequencing microarray for detection and characterization of botulinum neurotoxin-producing clostridia. PLoS One 8:e67510. doi: 10.1371/journal.pone.0067510, PMID: 23818983 PMC3688605

[ref41] Wu J. W. Cheng S. Zheng X. (1990). A report of an infant botulism case. Chin. J. Pediatr. 28:1.

[ref42] Xin W. Huang Y. Ji B. Li P. Wu Y. Liu J.. (2019). Identification and characterization of *Clostridium botulinum* strains associated with an infant botulism case in China. Anaerobe 55, 1–7. doi: 10.1016/j.anaerobe.2018.06.015, PMID: 30401636

[ref43] Zhang J. Gao P. Wu Y. Yan X. Ye C. Liang W.. (2022). Identification of foodborne pathogenic bacteria using confocal Raman microspectroscopy and chemometrics. Front. Microbiol. 13:874658. doi: 10.3389/fmicb.2022.874658, PMID: 36419427 PMC9676656

