# Supplementary figures and images for: Dissecting and tracing the gut microbiota of infants with botulism: a cross sectional and longitudinal study
Source: Front Microbiol. 2024 May 31;15:1416879. doi: 10.3389/fmicb.2024.1416879 (PMC11176563; doi:10.3389/fmicb.2024.1416879)

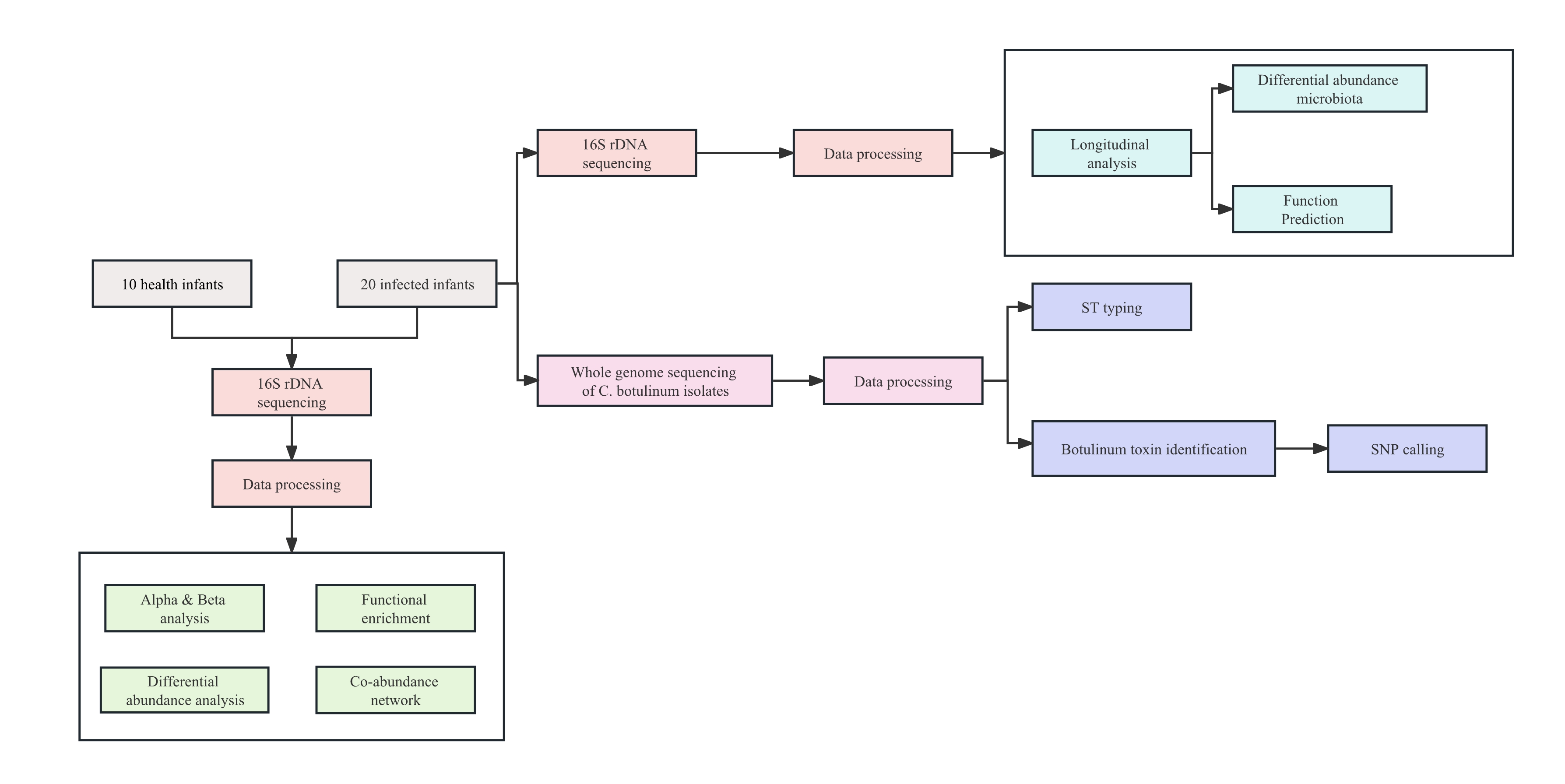

Supplement: Supplementary file 3 [file Image_1.JPEG]

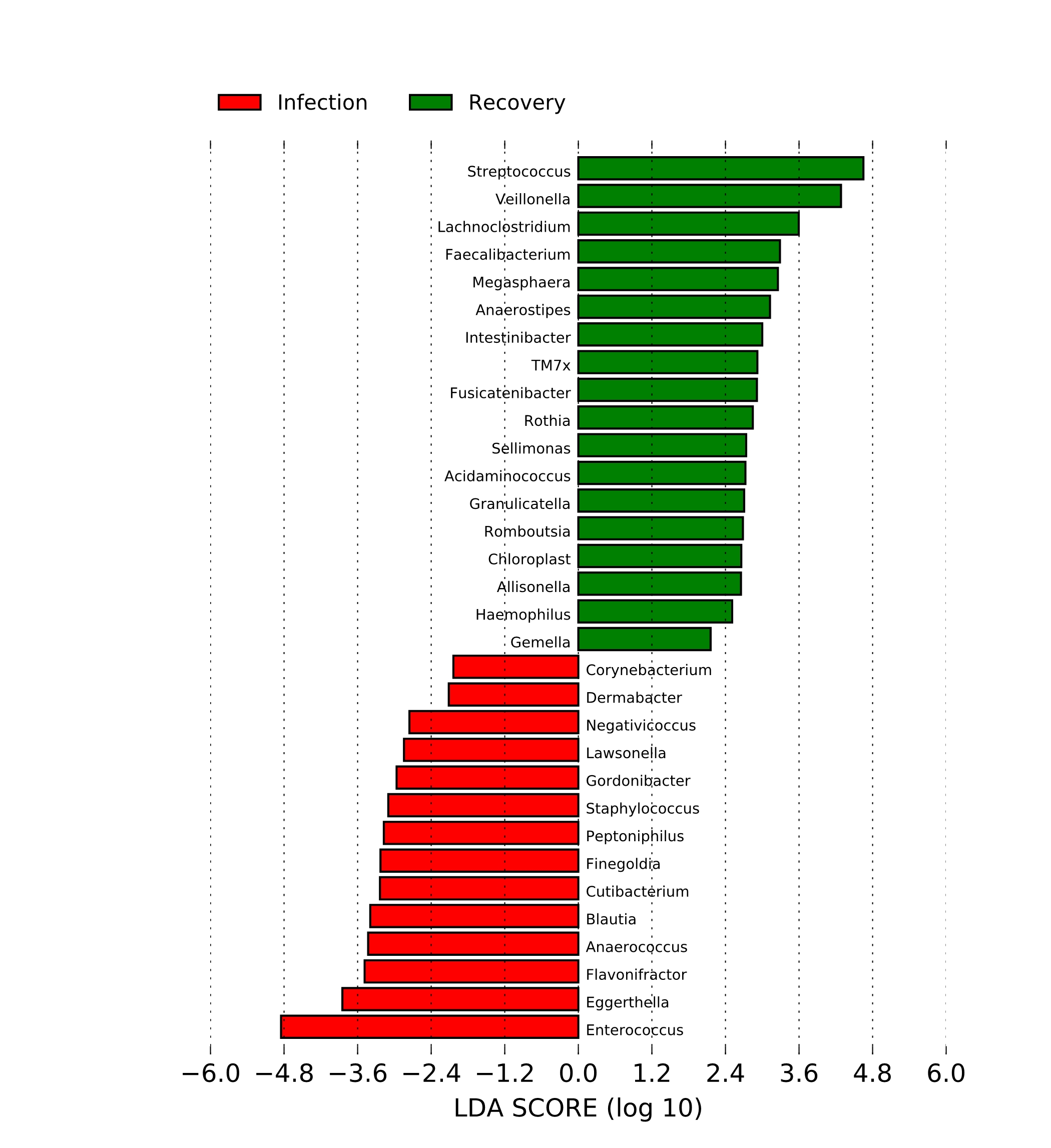

Supplement: Supplementary file 4 [file Image_2.JPEG]

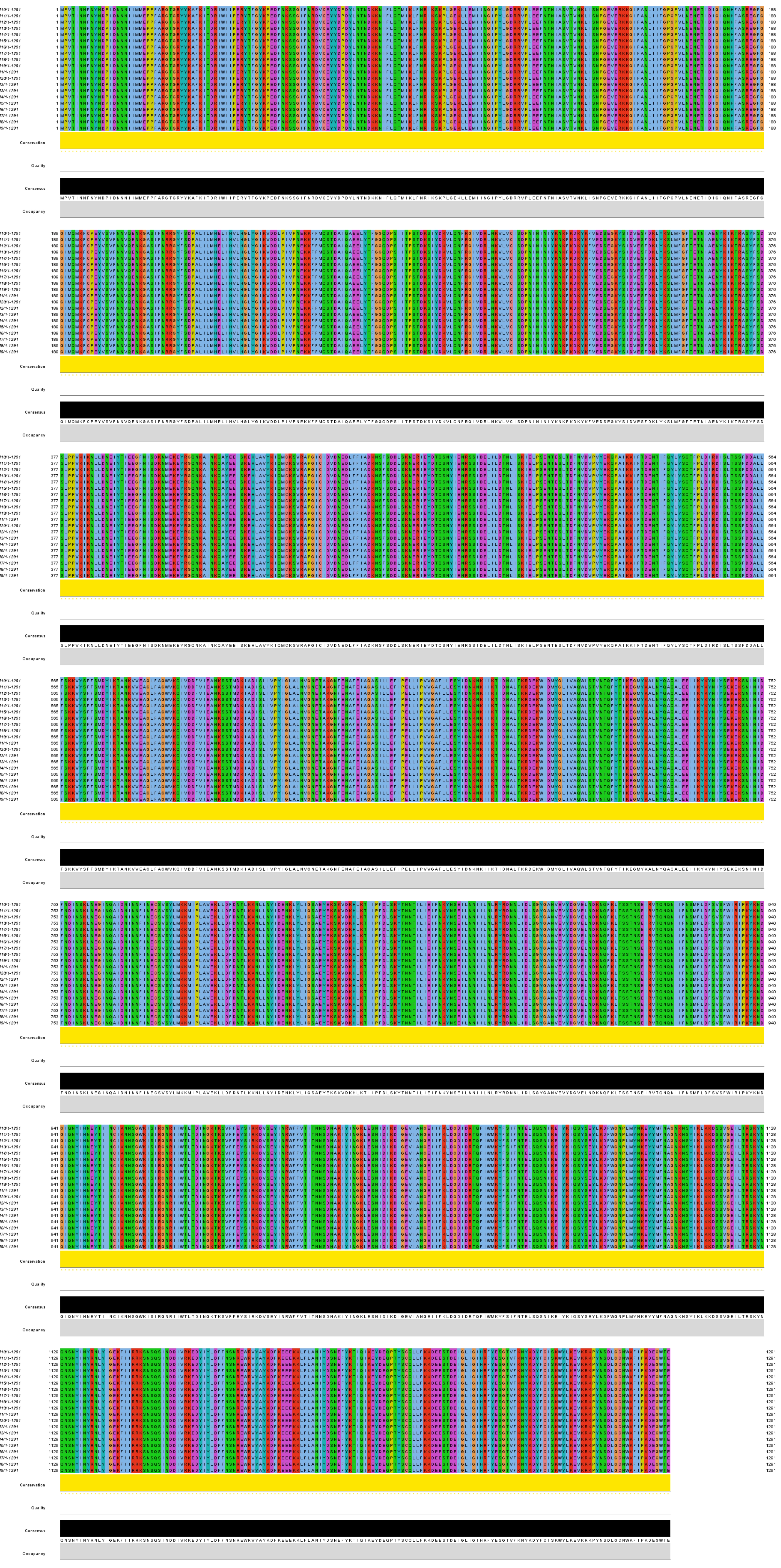

Supplement: Supplementary file 5 [file Image_3.JPEG]

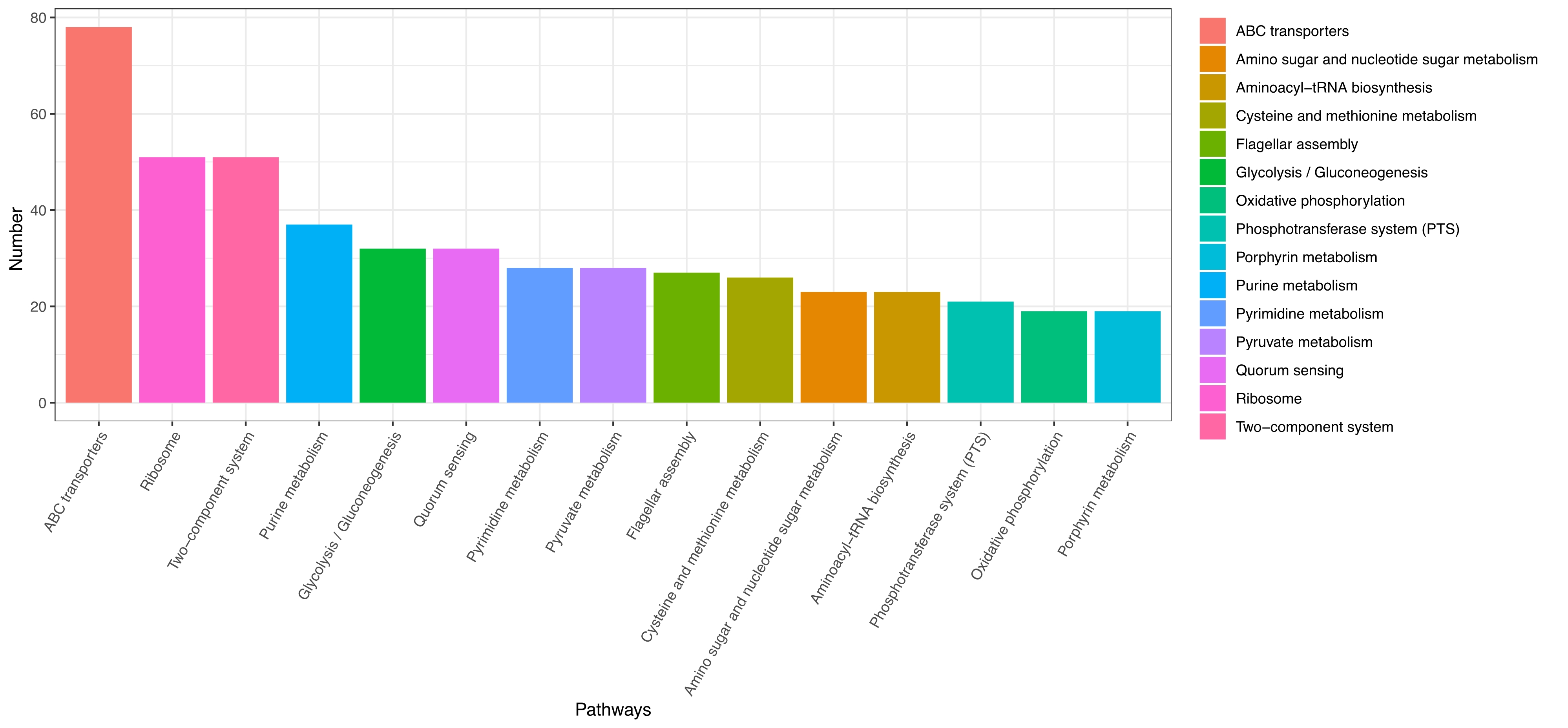

Supplement: Supplementary file 6 [file Image_4.JPEG]

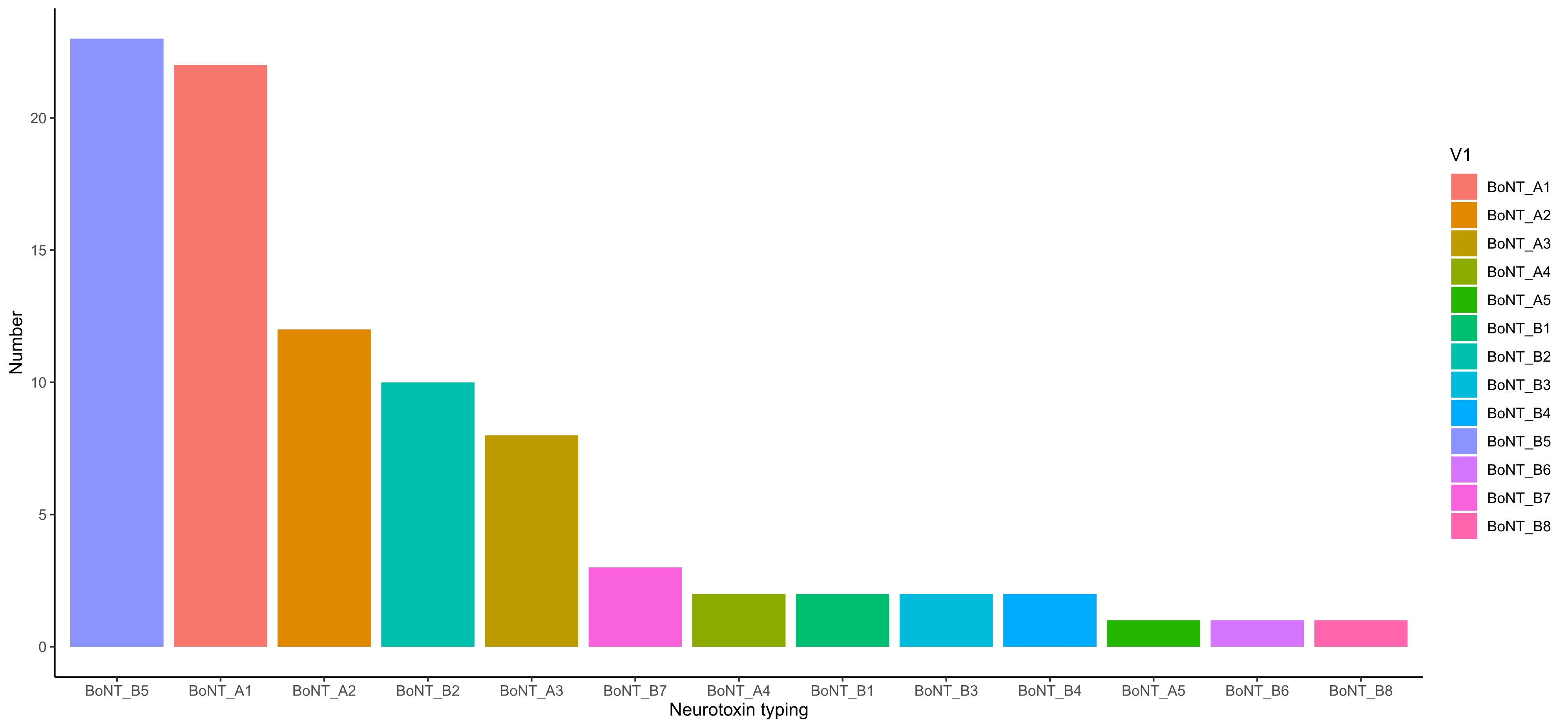

Supplement: Supplementary file 7 [file Image_5.JPEG]
